# Health Literacy and Its Related Determinants in Migrant Health Workers and Migrant Health Volunteers: A Case Study of Thailand, 2019

**DOI:** 10.3390/ijerph17062105

**Published:** 2020-03-22

**Authors:** Hathairat Kosiyaporn, Sataporn Julchoo, Pigunkaew Sinam, Mathudara Phaiyarom, Watinee Kunpeuk, Nareerut Pudpong, Rapeepong Suphanchaimat

**Affiliations:** 1 International Health Policy Program, Ministry of Public Health, Nonthaburi 11000, Thailand; sataporn@ihpp.thaigov.net (S.J.); pigunkaew@ihpp.thaigov.net (P.S.); mathudara@ihpp.thaigov.net (M.P.); watinee@ihpp.thaigov.net (W.K.); nareerut@ihpp.thaigov.net (N.P.); RAPEEPONG@IHPP.THAIGOV.NET (R.S.); 2 Division of Epidemiology, Department of Disease Control, Ministry of Public Health, Nonthaburi 11000, Thailand

**Keywords:** migrant health worker, migrant health volunteer, health literacy, Thailand

## Abstract

Migrant health workers (MHWs) and migrant health volunteers (MHVs) are key health workforce actors who play a substantial role in improving the health of migrants in Thailand. The objective of this study was to explore the factors associated with health literacy in MHWs and MHVs in Thailand. A self-administered questionnaire was conducted from December 2018 to April 2019 in two migrant-populated provinces. A total of 40 MHWs, 78 MHVs, and 116 general migrants were included in the survey. Results showed that a higher education level was associated with a greater health literacy score. MHWs were more likely to have a higher health literacy score (5.59 points difference) than general migrants. The province per se and type of affiliations did not significantly contribute to the difference in the health literacy score of each individual. Most MHWs received health information from health professionals, health staff, and the internet, while MHVs and general migrants received information from health professionals, MHWs/MHVs, family/friends, and posters/leaflets. This study suggests that a higher education level should be used as a criterion for recruitment of MHWs and MHVs. Access to interactive health information like health professionals should be promoted as the main source of information to ensure better health literacy among MHWs and MHVs.

## 1. Introduction

Effective health communication is a key component to achieve the safety and quality of healthcare of any population group, but it is much more crucial for people like migrants who have different language and cultural backgrounds to health personnel [1]. There are numerous barriers in health communication that migrants may encounter, including differences in language, culture, and health literacy between migrants (as service users) and health personnel (as providers) [1]. In some situations, family and friends can serve as ad-hoc interpreters and this practice is prone to communication errors and misdiagnosis, depending on their language ability and understanding of the issue. In addition, for some sensitive issues, patients may feel uncomfortable in disclosing their symptoms in front of their family members or friends [2]. To overcome these challenges, health facilities employ various strategies such as hiring bilingual health professionals and professional interpreters [3]. With a recognition of the importance of cultural and language differences, formal interpreters should not only translate the health message as it stands, but also consider the meaning of words that match migrants’ social and cultural contexts [2]. Interpreters who are familiar with migrants’ backgrounds are more likely to interpret or convey messages better than those who do not. Therefore, recruiting migrants to serve as interpreters or as health educators is an effective strategy to overcome communication barriers [4]. For example, in the United Kingdom, the Netherlands, Belgium, Spain, France, and Italy, there is a recruitment of migrants from the communities to serve as ‘cultural mediators’ who provide a link between health professionals and migrant patients [3]. 

In Thailand, an initiative called “migrant-friendly services” has been operating since 2003. The major component of the initiative is to recruit migrants to serve as migrant health workers (MHWs) and migrant health volunteer (MHVs). The MHW and MHV programmes were implemented by the Thai Ministry of Public Health (MOPH) in collaboration with a group of non-governmental organisations (NGOs) [5]. MHWs are assigned to be interpreters or health assistants in health facilities or NGOs and are required to collaborate and educate MHVs and migrant populations in particular areas. MHWs are hired by these organisations, while MHVs undertake volunteer work. MHVs are mostly responsible for working with migrants in their communities rather than in health facilities. MHVs are assigned to serve as community health educators and coordinators [6,7]. 

MHW and MHV programmes are an integral part of “migrant-friendly services” supported by the Ministry of Public Health (MOPH) through national curriculum manuals. The manuals address selection criteria as well as training and supervision processes for MHWs and MHVs [6,7]. MHWs and MHVs are required to have similar backgrounds to general migrants, to have good communication skills in both origin and destination countries’ languages, show leadership and the spirit of volunteerism, and be acceptable to the communities. In addition, both MHWs and MHVs are required to attend a training course provided by either the public sector such as provincial health office or health facilities or NGOs. MHWs are trained by health staff while MHVs are trained by both health staff and MHWs. The training courses were designed based on national curriculum manuals, organisational objectives and local contexts. Moreover, the MOPH and NGOs also provide a supporting system to generate a good working environment for MHWs and MHVs such as allowances and benefits for MHWs and non-financial incentives for MHVs. 

Health literacy is an ability to access, understand, appraise, and apply health information in order to make decisions for personal, family, and community health gains [8]. Migrants are classified as a vulnerable group, who are more likely to have low health literacy levels with limited access to health services and poor medical compliance, which usually leads to poor health outcomes [9,10]. 

It is expected that MHWs and MHVs should have adequate levels of health literacy; they are key people who play an active role in health communication and in promoting a healthy environment in the communities. There are many factors associated with health literacy which include both individual and system determinants such as demographic, psychosocial, sociocultural, education, and health system factors [8]. 

Academic studies have previously explored health literacy levels and their determinants in migrants [11]. A systematic review by Sørensen et al (2012) found that age, gender, race, socioeconomic status, culture and language backgrounds, functional and numerical literacy skills, social media use, social support, as well as environmental and political forces all affected health literacy levels [8]. Another systematic review on migrants mentioned additional factors related to health literacy such as acculturation, duration of residence in the destination country, and fluency in the primary language [11].

However, little is known about the factors that may affect the health literacy levels of MHWs and MHVs in comparison with migrants in general in Thailand. This study therefore aimed to assess the health literacy levels of all these groups and identify the factors that may influence them by using Thailand as a case study. 

## 2. Methods

### 2.1. Scope of the Study

**Figure 1:**
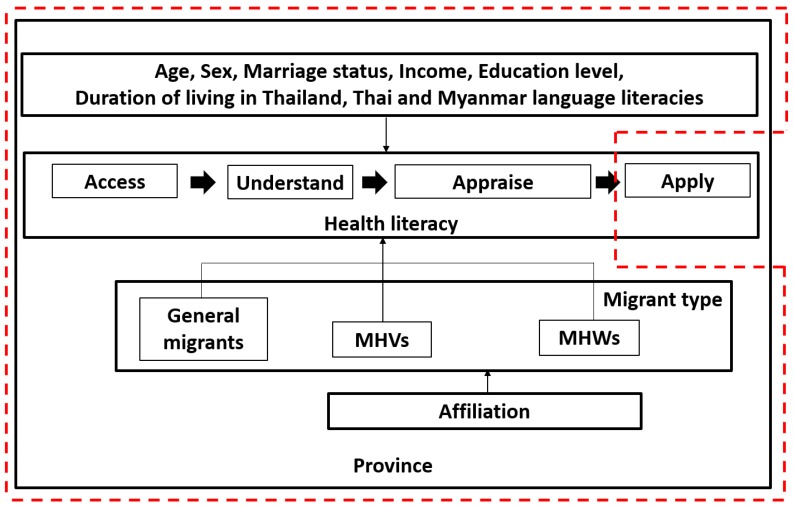
Conceptual Framework of associated factors with health literacy. Red line is the scope of this study.

Health literacy consists of four key components which are to access, understand, appraise, and apply health information [8]. However, the scope of this study focuses on three components of health literacy, namely, accessibility, understanding, and appraisal on health information. The application of health information to make a decision is excluded due to multifactorial aspects, which make it difficult to assess this area within a limited timeframe. The associated factors with health literacy in MHWs and MHVs were selected as interested variables, see Figure 1. 

### 2.2. Study Design

This study employed a quantitative approach and was conducted from December 2018 to April 2019. Provinces A and B were purposively selected (the real name of provinces is blinded to protect the participants’ confidentiality) as they are among the topmost highly migrant-populated provinces in Thailand. In each province, the headquarter district (Amphoe Muang) was used as the study site because they were the most densely populated with migrants. Ethics approval was obtained from the Institute for the Development of Human Research Protections, Thailand (IHRP 530/2561).

### 2.3. Participant Selection

The sample size was calculated by using two-sample parallel proportions with equality formula; see supplementary material 1. With such a formula, a total 120 of MHWs/MHVs had to be selected. Due to the limited number of active MHWs and MHVs in the two selected provinces, all currently active MHWs and MHVs were recruited. Owing to the key role in operating the MHV programme, MHVs under public provision were recruited in province A while those under NGO provision were recruited in province B. The researchers then recruited 120 general migrants who lived in the neighbourhood of each selected MHWs/MHVs (1:1 neighbourhood matching). Note that the term general migrants in this study refers to all cross-border migrants regardless of their immigration status (either documented or undocumented), occupations and nationality, who were neither MHWs nor MHVs. As the study sites are mainly Burmese-populated provinces, the majority of participants were Burmese (94.44%) who mostly work in the informal sector (30.77%).

### 2.4. Questionnaire Design

A self-administered questionnaire survey was performed. For the respondents who had difficulty in reading and understanding texts, a face-to-face interview with interpreters supported the completion of the questionnaire. The questionnaire was adapted from Osborne et al (2013) and Intarakamhang (2017) and was piloted among 24 migrant participants. A reliability test was performed. The Cronbach’s alpha coefficient equated 0.8920, representing a high level of reliability. In the pilot test, most of the respondents were married female, aged from 21 to 40 years, and graduated from secondary school. Most of them had a daily income lower than the minimal wage standard set by the Government (US$ 11 in province A and US$ 10 in province B). There were 27 questions in total comprising demographic data including self-rated literacy skills in Thai and Myanmar languages (Section 1: 14 questions), self-rated health literacy score (Section 2: 12 questions) and channels through which MHW/MHV/general migrants receive health information (Section 3: 1 question). The surveys in MHWs and MHVs were conducted by a research team at health facilities or NGOs, whereas the surveys on general migrants were delivered by MHWs at migrant communities. The MHWs who conducted the surveys were trained by a research team before start of working. 

### 2.5. Data Analysis and Variable Management

Data were made anonymous by using a code number to identify each respondent, instead of their names. Data curation and analysis was performed by HK and SJ in secured computers. Descriptive statistics were used in all sections of the questionnaire. Then the researchers used inferential statistics to assess the association between health literacy scores (Section 2) and independent variables, which were adopted from Section 1 of the questionnaire. These independent variables or demographic data were sex (male and female), age (years) (<20, 21–40, and 41–60), marriage status (single, married, and divorced/widowed), education level (no qualifications, primary school, secondary school, and university and above), income (lower than minimal wage and equal and higher than minimal wage), duration of living in Thailand (year) (≤5, 6–10, 11–15, and >15) and Thai and Myanmar language literacies (incapable, fair, proficient), migrant type (MHWs, MHVs and general migrants), affiliation, and province. The questions about health literacy were structured in 12 sub-questions and each sub-question was constructed as a Likert scale, ranging from 1 to 3 (1 means cannot do/disagree and 3 means easy/agree). The list of questions for the health literacy score is presented in supplementary material 2 (Table S1). The sum of the health literacy score (min 12 and max 36) served as the main dependent variable. For Section 3, which is about the acquisition of health information, respondents were asked to answer the three most preferable channels of health information in their opinion.

In detail, mean and percentage were used in descriptive statistics. For the inferential statistics, the analysis was divided into two parts: first, bivariate analysis and multivariable analysis. For the bivariate analysis, the researcher opted to use the t-test and ANOVA. For multivariable analysis, multiple regression was performed. Only significant association variables from bivariate analysis were used as inputs in the multivariable analysis at a cut point of p-value <0.05. 

In addition to the multivariable analysis, the researchers also performed a random-intercept model to assess whether the findings would change if the province variable acted as a supra- individual level variable rather than serving as an independent variable. All analyses were performed by STATA14 (StataCorp LP, Texas, TX, USA).

## 3. Results

### 3.1. Participants

A total of 234 respondents participated in this survey (~97.5% response rate). The participants comprised 40 MHWs, 78 MHVs, and 116 general migrants. There were nearly an equal number of samples from both provinces accounting for 110 in Province A (MHWs = 22, MHVs = 41, general migrants = 47) and 124 in Province B (MHWs = 18, MHVs = 37, general migrants = 69). Most of them were married females, aged from 21 to 40 years. More than half of them had completed secondary school education (about 60.00% of MHWs, followed by 50.00% and 49.14% in MHVs and general migrants, respectively). The daily income of MHWs was generally an equal or higher income than the minimal wage. In contrast, most MHVs and general migrants received daily remuneration less than the minimal wage. MHWs had a longer duration of living in Thailand (about 11–15 years) compared to general migrants (mostly shorter than 10 years). There were nearly equal numbers of samples in each type of affiliations (60 with public hospitals and 58 with NGOs). 

Literacy skills in reading and listening were evaluated in both Thai and Myanmar languages. There were obvious differences in abilities to listen and read the Thai language among MHWs, MHVs, and general migrants; MHWs had greater Thai-listening and -reading literacy skills than the other two groups, whereas other skills and abilities did not differ that much. A number of 77.50% of MHWs and 18.97% of general migrants were at proficiency level in listening to the Thai language while 50% of MHWs and 7.76% of general migrants were at proficiency level in reading the Thai language.

### 3.2. Health Literacy Score 

The results showed that the health literacy scores were nearly the same in all sexes, ages, married statuses, and income levels. The proportion of MHWs/MHVs that had a favourable health literacy level was 77.12% (more than 60% of the total score) while that of general migrants was 15.52%. Respondents who had completed at least university level education had a higher literacy score (30.88 ± 4.41) compared with those who were uneducated (21.00 ± 4.29). Moreover, the respondents who had lived in Thailand for more than ten years had higher health literacy scores than those who had lived in Thailand for less than ten years. The health literacy scores were found to be higher among respondents who were proficient in listening and reading skills, compared with those who had fair or had incapable literacy skills, especially in the Thai language. See Table 1.

There were no significant differences in health literacy scores between provinces, and affiliations. Health literacy scores in MHWs (30.38 ± 3.84) were higher than in MHVs (28.13 ± 4.42) and general migrants (21.23 ± 5.22). 

### 3.3. Channels through which MHW/MHV/General Migrants Receive Health Information

**Figure 2:**
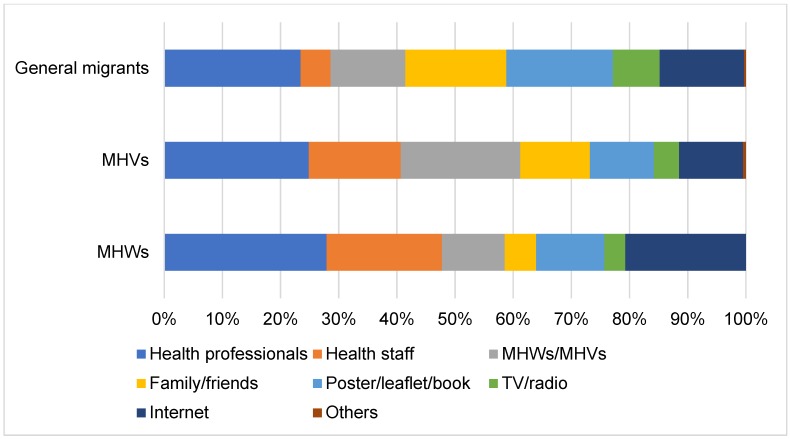
Channel of health information classified by types of migrants. (maximum three answers by each respondent). MHWs: migrant health workers; MHVs: migrant health volunteers.

Figure 2 demonstrates the channels through which study participants receive health information. It was found that MHWs receive health information mostly through health professionals and health staff, followed by the Internet. MHVs and general migrants also receive information mostly through health professionals, but the second most common channels were family members and friends, followed by posters and leaflets.

### 3.4. Factors Associated with Health Literacy Score

After conducting bivariate analysis, it was found that education level, duration of living in Thailand, Thai reading and listening skills, migrant type and affiliation were associated with the health literacy score. See supplementary material 3 (Table S2). Respondents who had higher education levels tended to report greater health literacy scores. Respondents who had lived in Thailand for a longer period were more likely to have higher literacy scores. The more fluent the participant was in listening and reading the Thai language, the greater the health literacy score. MHWs were more likely to have higher health literacy scores compared with MHVs and general migrants. Respondents who were affiliated with NGOs tended to have better literacy scores than those with public organisations. 

All significant variables were used in the multivariate analysis. Although the province was not directly associated with the health literacy score in the bivariate analysis, it was defined as a higher hierarchical variable, which potentially had influence over other demographic determinants. Therefore, the province variable was always included in the multivariable analysis regardless of other factors.

Results from the multivariable analysis showed that province B had lower health literacy scores than province A by around 2.00 points. General migrants had lower health literacy scores than MHWs by approximately 5.59 points. Respondents who graduated from secondary school and university had respectively 4.24 and 5.25 points higher scores than those without an educational background. See Table 2.

The sensitivity analysis, using a random-intercept model where the province served as a supra- individual variable, was performed. See Table 3. The results showed a slight difference from the multiple regression model. This finding implies that the province per se did not significantly influence the health literacy score of each individual. 

## 4. Discussion

This study found that education levels were significantly associated with health literacy levels after adjusting for all potential confounders such as age, sex, marital status, income, duration of living in Thailand, and Thai and Myanmar language literacies. This finding is consistent with a previous study conducted in the US, which showed that education attainment was associated with health literacy levels [12]. This suggests that the recruitment of new MHWs and MHVs should focus on migrants with some levels of education attainment.

The MHW and MHV programmes are hugely beneficial to the health service system in Thailand. Their merits are evident in various ways; for instance, reducing language and cultural barriers, enabling health professionals to serve hard-to-reach migrants, and promoting migrant-friendly services [4]. Though the Thai Government expresses strong support for these programmes, there is still room for improvement, particularly in terms of increasing health literacy among MHWs and MHVs. Health literacy is an important factor that leads to effective health communication [2]. To strengthen health literacy in MHWs and MHVs, it is necessary to understand the associated factors that determine existing levels of health literacy.

A systematic review by Fernández-Gutiérrez (2018) on health literacy interventions suggested that access to health resources was a cornerstone for achieving better health in the wider population [13]. Moreover, the National Statement on Health Literacy 2016 by the Australian Commission on Safety and Quality in Healthcare proposed that to improve health literacy, people should regularly be engaged in health education and have opportunities to discuss issues of concern with healthcare providers [14]. Interactive health communication with health professionals, health staff, and social media should be considered as key sources of health information.

Note that health literacy scores in MHWs were apparently higher than in MHVs and general migrants. This finding suggests that the overall capacity building process of the MHW programme helps to increase the health literacy levels of the trainees. According to results about health information channels, one possible explanation of this success is that MHWs generally have greater exposure to health information directly from health professionals and health staff because most of the MHWs worked in the health facilities and NGOs. In contrast, MHVs and general migrants tended to receive health information through informal means, such as family members, friends, and posters and leaflets.

A reliance on families and friends is another source of health information. A previous study by the World Health Organization (WHO) Regional Office for Europe (2010) reported that some migrants preferred to use the networks in their communities to gain health information because of trust and the sharing of similar backgrounds [3]. This point has policy implications which are that health messages should not be delivered by mainstream health professionals or health staff only. Messages should be imbued into migrant communities and MHVs could be commissioned to perform this function. 

Another point worth mentioning is that from the multiple regression, province A (Central region) participants had a higher literacy score than those in province B. In the random-intercept model where the province acted as a higher hierarchical variable, the results did not change much from the multiple regression model. This alludes to the fact that there might be some intrinsic and unobserved characteristics of the province variable (which are independent of the characteristics of an individual) that might affect the literacy level of the participants. Such factors might be the features of the MHW/MHV programme which can vary by province (as each province has some degree of discretion to operate the programme in its own way). This might include the difference in training duration and course content, the intensity and frequency of arranging a refreshing course and the provision of other supporting learning materials. Further studies (likely to be qualitative in nature) aiming to explore how each province operates the MHW/MHV programme are of great value to address this issue [15]. 

To the best of the authors’ knowledge, this study is probably one of the first studies in Thailand that systematically evaluates health literacy levels and related determinants among MHWs and MHVs, in comparison with general migrants. Though design was rigorous, there remained some limitations. First, findings of this study may not be generalizable throughout the country because the study sites were selected by a purposive sampling. Second, the assessment was conducted only from the MHWs’ and MHVs’ perspective through a self-reporting questionnaire. A more rigorous assessment of health literacy of migrants is therefore recommended such as evaluation of migrants’ health literacy based on health personnel perspectives or other methodologies to triangulate the data. Third, although interpreters who assisted in conducting the surveys received the explanations about the meaning of each question, there is an opportunity of misinterpretation that influences the validity of data. Finally, there might be other confounders that influence health literacy levels, but which were not evaluated in this study, such as sociocultural factors and learning processes and methods. Further studies that investigate this matter further are recommended. 

## 5. Conclusions

MHWs and MHVs are key health cadres in the Thai health care system. They significantly help reduce language and cultural gaps between health personnel and migrants. Overall, MHWs had greater health literacy levels than MHVs and general migrants. Educational background is the key determinant that leads to better health literacy levels and this might be considered as one of the criteria in recruiting new cadres of MHWs and MHVs. In terms of health information access, MHWs who have greater health literacy, mostly rely on health professionals and health staff, whereas MHVs and general migrants tend to rely more on migrant networks, as well as posters and pamphlets. This means that the relay of health information should not be done only in health facilities but should also be embedded in migrant communities. Further studies that examine the actual operations of the MHW/MHV programme in different provinces and focus on sociocultural factors and learning processes are recommended. This would help gain a better understanding of the entire MHW/MHV programme in Thailand.

## Figures and Tables

**Table 1:** Health literacy score of respondents classified by demographic data.

Variables		Number	Health Literacy Scores	
			Mean (SD)	Median (Min–Max)
Province	A	110	25.81 (5.61)	26 (15–36)
	B	124	24.46 (6.53)	23.5 (14–36)
Migrant type	MHWs	40	30.38 (3.84)	30.5 (22–36)
	MHVs	78	28.13 (4.42)	29 (16–36)
	General migrant	116	21.23 (5.22)	20 (14–36)
Sex (n = 233)	Male	81	25.12 (6.14)	25 (15–36)
	Female	152	25.11 (6.01)	24 (14–36)
Age (years)	<20	17	25.71 (7.22)	26 (14–36)
	21–40	153	24.80 (6.10)	24 (15–36)
	41–60	64	25.64 (5.97)	26 (12–36)
Married status (n = 230)	Single	55	26.78 (6.10)	29 (14–36)
	Married	162	24.65 (6.09)	24 (15–36)
	Divorced/widowed	13	25.54 (5.58)	24 (18–35)
Education levels (n = 223)	No qualifications	6	21.00 (4.29)	19.5 (16–28)
	Primary school	81	22.72 (6.26)	21 (12–36)
	Secondary school	120	26.07 (5.61)	26 (16–36)
	University and above	16	30.88 (4.41)	31.5 (22–36)
Income/Month (n = 229)	Lower than minimal wage *	129	24.57 (6.58)	24 (14–36)
	Equal or higher than minimal wage *	100	25.8 (5.54)	25 (15–36)
Duration in Thailand (years) (n = 227)	≤5	53	22.72 (5.25)	23 (14–36)
	6–10	71	24.13 (6.16)	24 (12–36)
	11–15	49	27.08 (6.27)	28 (15–36)
	>15	54	26.24 (5.98)	27 (15–36)
Myanmar listening skills (n = 225)	Incapable	9	26.33 (5.81)	26 (15–36)
	Fair	15	23.07 (5.02)	23 (15–33)
	Proficient	201	25.15 (6.23)	24 (14–36)
Myanmar reading skills (n = 232)	Incapable	9	22.78 (6.53)	19 (15–33)
	Fair	39	25.33 (6.89)	26 (12–36)
	Proficient	184	25.15 (5.99)	24 (14–36)
Thai listening skills (n = 232)	Incapable	40	23.13 (4.84)	23.5 (15–36)
	Fair	117	24.19 (6.22)	24 (14–36)
	Proficient	75	27.43 (6.00)	29 (16–36)
Thai reading skills (n = 233)	Incapable	138	23.14 (5.78)	22.5 (14–36)
	Fair	55	27.36 (5.52)	28 (14–36)
	Proficient	40	28.73 (5.58)	30 (15–36)
Affiliation (n = 118)	Public hospitals	60	28.32 (4.96)	29 (16–36)
	NGOs	58	29.48 (3.56)	29 (22–36)
**Total**		**234**	**25.09 (6.14)**	**24.5** (14–36)

* Minimal wage in province A is 325 Baht (US$ 11) and province B is 310 Baht (US$ 10). MHW: migrant health worker; MHV: migrant health volunteer; SD: standard deviation; NGO: non-governmental organisations.

**Table 2:** Factors associated with health literacy score from multiple regression.

Demographic Data		Number	Coefficient	P-Value	95% CI
Province	A	111	Reference		
	B	124	−2.00	0.009	−3.50–(−0.51)
Migrant type	MHWs	40	Reference		
	MHVs	78	0.06	0.954	−2.14–2.27
	General migrants	116	−5.59	<0.001	−8.26–(−2.93)
Education level	No qualifications	6	Reference		
	Primary school	81	1.56	0.429	−2.32–5.44
	Secondary school	120	4.24	0.032	0.38–8.11
	University and above	16	5.25	0.033	0.42–10.08
Duration in Thailand (years)	≤5	53	Reference		
	6–10	71	−0.31	0.729	−2.07–1.45
	11–15	49	0.63	0.547	−1.43–2.69
	>15	54	0.52	0.616	−1.53–2.57
Thai listening level	Poor	40	Reference		
	Fair	117	0.97	0.311	−0.92–2.86
	Good	75	0.31	0.799	−2.06–2.67
Thai reading level	Poor	138	Reference		
	Fair	55	1.17	0.176	−0.53–2.87
	Good	40	2.16	0.071	−0.19–4.51
Affiliation	Public hospitals	60	Reference		
	NGOs	58	1.13	0.301	−1.02–3.29

Missing data were excluded as the number of missing records were negligibly low. MHW: migrant health worker; MHV: migrant health volunteer; NGO: non-governmental organisations; CI: confidence interval.

**Table 3:** Random-intercept model on health literacy score.

Demographic Data		Number	Coefficient	SD	P-Value	95% CI
Migrant type	MHWs	40	Reference			
	MHVs	78	−0.04	1.08	0.974	−2.15–2.08
	General migrants	116	−5.96	1.28	<0.001	−8.47–(−3.45)
Education level	No qualifications	6	Reference			
	Primary school	81	1.40	1.90	0.462	−2.33–5.12
	Secondary school	120	3.97	1.89	0.036	0.27–7.67
	University and above	16	4.95	2.36	0.036	0.32–9.57
Duration in Thailand (years)	≤5	53	Reference			
	6–10	71	−0.28	0.86	0.744	−1.97–1.41
	11–15	49	0.58	1.01	0.564	−1.40–2.56
	>15	54	0.36	1.00	0.722	−1.60–2.31
	Poor	40	Reference			
Thai listening level	Fair	117	1.09	0.92	0.239	−0.72–2.90
	Good	75	0.43	1.16	0.710	−1.84–2.70
	Poor	138	Reference			
Thai reading level	Fair	55	1.14	0.83	0.171	−0.49–2.78
	Good	40	2.13	1.15	0.064	−0.13–4.39
Affiliation	Public hospitals	60	Reference			
	NGOs	58	0.85	1.04	0.414	−1.19–2.89

Estimated variance of intercept (province) showed P-value = 0.0716. MHW: migrant health worker; MHV: migrant health volunteer; NGO: non-governmental organisations; SD: standard deviation; CI: confidence interval.

## Supplementary Materials

The following are available online at https://www.mdpi.com/1660-4601/17/6/2105/s1, Table S1: Health literacy questionnaire, Table S2: The results of bivariate analysis.
