# Supplementary material for: Health Literacy and Its Related Determinants in Migrant Health Workers and Migrant Health Volunteers: A Case Study of Thailand, 2019
Source: Int J Environ Res Public Health. 2020 Mar 22;17(6):2105. doi: 10.3390/ijerph17062105 (PMC7143383; doi:10.3390/ijerph17062105)
Supplement: Supplementary file 1 [file ijerph-17-02105-s001.pdf]

Supplementary Materials

Supplementary material 1

Formula of sample size calculation

n = $2[Z_{\alpha/2} + Z_{\beta}]^2\sigma^2/d^2$

The difference of mean score in two groups (d) equals to 3 [1]
The standard deviation in unexposed group (σ) equals to 5.23 [1]

$Z_{\alpha/2}$ equals to 1.96 at 95% of confidence interval.
Z_{β} equals to 0.84 at 80% of discrimination power.

When the formula was calculated, there were 48 samples for each province. Considering a possible 20% non-response rate, 60 samples were selected in each province or 120 for both provinces. Additionally, 120 general migrants in that area were recruited by using 1:1 matching strategy to compare as baseline data.

Supplementary material 2

Table S1. Health literacy questionnaire.

Question	Answer		
	**Cannot Do /Disagree (Score 1)**	**Difficult/Neutral (Score 2)**	**Easy/Agree (Score 3)**
1. I can find information about health problems or concerns			
2. I can find health information from different places e.g., health personals, media, etc.			
3. I always check health information in order to keep myself in good health			
4. I have enough health information to deal with my health problems			
5. After I have read health information in my native language, I can understand all information			
6. After I have heard health information in my native language, I can understand all information			
7. After getting health information, I try to understand the information			
8. After receiving health information, I always compare health information from different sources			
9. When I see new information about health, I always check whether the sources are reliable before I believe or follow.			
10. I know how to find reliable sources of health information before I believe or follow.			
11. You have a competency to make a right decision after receiving health information			
12. You have a competency to share health information to others			

Supplementary material 3

Table S2. The results of bivariate analysis.

Variables	P-value
Province	0.0520
Migrant type	<0.001 *
Sex	0.9983
Age	0.5201
Married status	0.0532
Income	0.0605
Education level	<0.001 *
Duration of living in Thailand	0.0021 *
Myanmar listening skill	0.3615
Myanmar reading skill	0.5161
Thai listening skill	<0.001 *
Thai reading skill	<0.001 *
Affiliation	<0.001 *

* P-value < 0.05.

Reference

1. Intarakamhang, U. *Creating and Developing of Thailand Health Literacy Scales*; Behavioral Science Research Institute, Srinakharinwirot University: Bangkok, Thailand, 2017.
